# Can the Full-Percutaneous Endoscopic Lumbar Discectomy in Day Surgery Mode Achieve Better Outcomes Following Enhanced Recovery after Surgery Protocol? A Retrospective Comparative Study

**DOI:** 10.3389/fsurg.2022.914986

**Published:** 2022-05-20

**Authors:** Le Kou, Wentao Wan, Chao Chen, Dong Zhao, Xun Sun, Ziwei Gao, Hongjin Wu, Mingyuan Di, Xinlong Ma, Baoshan Xu, Jun Miao, Zheng Wang, Qiang Yang

**Affiliations:** ^1^ Department of Spine Surgery, Tianjin Hospital, Tianjin University, Tianjin, China; ^2^ Department of Orthopedics, Tianjin Baodi Hospital, Baodi Clinical College of Tianjin Medical University, Tianjin, China; ^3^ Graduate School, Tianjin Medical University, Tianjin, China; ^4^ Department of Orthopedics, No.1 Medical Center of Chinese PLA General Hospital, Beijing, China

**Keywords:** percutaneous endoscopic lumbar discectomy, day surgery, enhanced recovery after surgery, lumbar disc herniation, endoscopy

## Abstract

**Background:**

Full-percutaneous endoscopic lumbar discectomy (F-PELD) is a popular operation for the treatment of lumbar disc herniation (LDH). Some studies have reported that F-PELD in day surgery mode produced favorable outcomes for LDH. At the same time, minimally invasive spinal surgery following enhanced recovery after surgery (ERAS) presents a rising trend in recent years, but few studies reported whether F-PELD will produce better outcomes in the day surgery (DS) mode combined with ERAS.

**Objective:**

To analyze whether F-PELD in day surgery mode following ERAS can produce better clinical outcomes than in traditional surgery mode.

**Methods:**

The patients who underwent F-PELD between January 2019 and October 2020 were retrospectively analyzed, and the patients who met the inclusive criteria were followed up. The patients were divided into day surgery (DS) group (*n* = 152) that combined with ERAS and traditional surgery (TS) group (*n* = 123) without ERAS. The length of hospital stays (LOS), visual analogue scale (VAS), and Oswestry Disability Index (ODI) of two groups were compared before surgery, immediately after surgery, one month after surgery, and one year after surgery.

**Results:**

A total of 298 patients who underwent F-PELD were reviewed. 290 patients were included in the study and followed up, and 275 patients who had completed the follow-up were available for analysis. There were no statistically significant differences between the two groups in terms of age, gender, preoperative VAS, and ODI. There were significant statistical differences in the VAS and ODI immediately after surgery (VAS for back pain: DS group 1.4 ± 1.1, TS group 2.0 ± 1.2, *p* < 0.001; VAS for leg pain: DS group 0.8 ± 0.8, TS group 1.1 ± 1.1, *p* = 0.010; ODI: DS group 5.8 ± 4.3, TS group 7.6 ± 7.4, *p* = 0.010) and one month after surgery (VAS for back pain: DS group 0.8 ± 0.9, TS group 1.1 ± 1.0, *p* = 0.035; ODI: DS group 3.2 ± 3.5, TS group 4.5 ± 6.5, *p* = 0.036). At one year after surgery, the VAS (back pain: DS group 0.3 ± 0.6, TS group 0.3 ± 0.7, *p* = 0.798; leg pain: DS group 0.2 ± 0.4, TS group 0.1 ± 0.4, *p* = 0.485) and ODI (DS group 0.8 ± 1.2, TS group 0.7 ± 1.7, *p* = 0.729) were further improved, but no statistically significant difference was observed between two groups. LOS of DS group (1.38 ± 0.49 days) was significantly shorter than the TS group (5.83 ± 2.24 days, *p* < 0.001), and some postoperative complications occurred in the TS group, including throat discomfort (*n* = 5, 4.1%), discomfort after catheterization (*n* = 7, 5.7%), abdominal distention (*n* = 3, 2.4%), and nausea (*n* = 5, 4.1%). None of the above complications resulted in serious consequences.

**Conclusion:**

The F-PELD in day surgery mode following ERAS produced a better short-term clinical effect and reduced the LOS, which is worthy of promotion.

## Introduction

Lumbar disc herniation (LDH) is one of the most common lumbar intervertebral disc degenerative diseases. Patients with the LDH often seek medical attention for discogenic low back pain or radiating pain, most of which are caused by herniated discs pressing on nerves (1, 2). Percutaneous endoscopic lumbar discectomy (PELD) has recently become popular in the treatment of LDH due to the smaller skin incision, light tissue damage, shorter operation time, rapid recovery, and earlier return to work (3–5). At present, most studies show that the average length of stay of PELD as a traditional model is 2 to 5 days (6–8). However, some studies have shown that PELD in day surgery mode has also produced good clinical outcomes, and these researchers believe that day surgery is feasible for PELD (9). Therefore, the comparison of these two procedures has great clinical significance.

Enhanced recovery after surgery (ERAS) is a clinical concept based on evidence-based medicine, optimizes clinical pathways of perioperative management through multidisciplinary collaboration in surgery, anesthesia, nursing, and nutrition to reduce postoperative complications, shorten hospital stay, and promote recovery (10, 11). ERAS has been widely used in colorectal, joint surgery (12, 13). In recent years, some studies have reported that the application of ERAS in spinal surgery also produced obvious advantages (14–16). However, the use of ERAS in F-PELD has rarely been reported, and the specific protocol of ERAS has not been formulated. It is not clear whether F-PELD following the day surgery (DS) mode, which combined with ERAS, will produce better effects. Therefore, this study aims to evaluate the clinical outcomes of F-PELD in day surgery mode following ERAS and to provide some relevant clinical evidence for the treatment of LDH.

## Materials and Methods

### General Information

From January 2019 to October 2020, 298 patients with LDH were treated with F-PELD at the Spinal Surgery department of Tianjin Hospital, and the patients who met the criteria for inclusion and exclusion were included in the follow-up. They were divided into the day surgery (DS) group, combined with ERAS and traditional surgery (TS) groups. All surgeries were performed by the same surgeon. The length of hospital stay (LOS), low back and leg pain visual analogue scale (VAS), and Oswestry Disability Index (ODI) were compared between two groups.

The inclusion criteria are as follows:

(a) The diagnosis is consistent with the clinical and imaging examinations,
(b) The primary spinal endoscopic surgery,
(c) Patients who have been ineffective for more than three months after receiving conservative treatment.

The following exclusion criteria are applied:

(a) Previous lumbar spine surgery,
(b) Mental illness or cognitive dysfunction,
(c) Other comorbidities or severe systemic diseases such as tumors, gout, and infections.

The study was approved by the hospital ethics committee.

### Surgery Procedure

All operations were performed by the same surgeon who had many years of experience in the PELD technique.

(a) For the patients who underwent F-PELD in day surgery mode, we provided the preoperative education in order to alleviate the scared and anxious feelings of them. Patients were given a nonsteroidal anti-inflammatory analgesic (celecoxib 100 mg) and pain threshold raising drugs (pregabalin 75 mg) two hours before surgery. And Midazolam (0.5–1 mL) was injected intramuscularly right before operation. patients with L5/S1 disc herniation were positioned prone, while patients with L3/4 and L4/5 disc herniation were positioned laterally. We used a C-arm x-ray to define the entry spot before puncturing. The surgical area was disinfected, and local anesthesia was performed at the entry point. Then, an 18-G needle was used to anesthetize the path with 1% lidocaine. A small number of patients undergoing surgery with local anesthesia may experience unbearable intraoperative pain, which can be increased by adding ropivacaine to increase the anesthetic effect. If we found foraminal stenosis under endoscopy, we could remove part of the bone on the ventral side of the superior articular process by using a trephine. If the patient is in severe pain, lateral access nerve block anesthesia should be used in addition to the anesthetic methods described above. And then, the following procedure was surgery. During the operation, straight leg raising test was performed to detect the surgical effect because patient who underwent the individual local anesthesia was in a conscious state. At the end of the surgery, the dural sac and nerve-root were freely mobilized. Betamethasone (4 mg) was administered to the local nerves before the wound was closed. After the surgery, patients took celecoxib and pregabalin orally to prevent the occurrence of pain, while topical analgesic plaster around the surgical area were used for postoperative analgesia. When patients went back to the ward, doctor would teach patients to perform rehabilitation exercises such as straight-leg-raising movement, ankle pump exercise, toe flexion, and extension. On the second day after surgery, the patient could wear a waist protector and ground exercise and doctor would make an individual excise plan for each patient according to their situation.
(b) For the patients who underwent F-PELD in tradition surgery mode, one day or two preoperative preparation was required before surgery, with routine fasting and water fasting before surgery. General anesthesia with a laryngeal mask airway was also adopted during the operation to achieve sufficient analgesia, appropriate sedation and full muscle relaxation. Due to the general anesthesia, preoperative catheterization is required. Patients with L5/S1 disc herniation were positioned prone, while patients with L3/4 and L4/5 disc herniation were positioned laterally. Then patients were performed by the same surgeon in traditional F-PELD routine. Postoperative fasting for 6 h, cardiac monitoring, oxygen, intravenous flurbiprofen and rehydration were administered, and the urinary catheter was removed the day after surgery. Patients in TS had to be discharged for 2 to 3 days after surgery to make sure they did not suffer from postoperative complications such as throat discomfort, discomfort after catheterization, abdominal distention and nausea.

### Advantages of F-PELD in Day Surgery Mode Following ERAS

**Figure 1:**
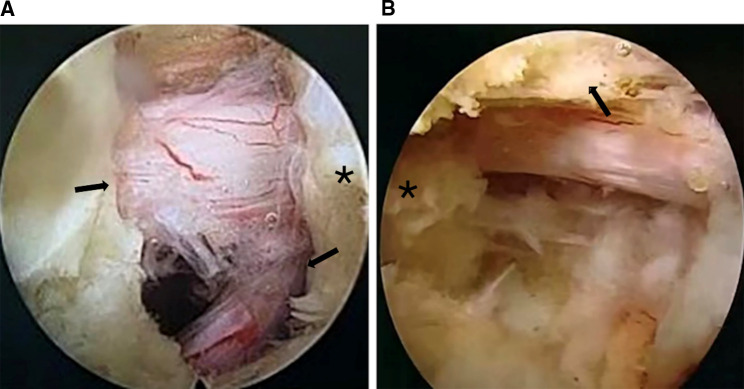
The intraoperative decompressed dural sac and nerve-root. (**A**) F-PELD via the posterior interlaminar approach in L5-S1, variant nerve root(see arrows), cranial side(see star). (**B**) F-PELD via the lateral transforaminal approach in L4-5 showed in the right picture, ventral side of the superior articular process (see arrows), cranial side(see star).

(a) Preoperative education: The primary goal of preoperative education is to calm patients’ nerves and anxiety, which greatly embodies the concept of Bio-Psycho-Social medical model. The surgeon should explain the surgery procedure, duration, possible surgery-related discomfort, postoperative rehabilitation exercise methods, and answer any questions from the patients. During the perioperative period, they kept the patients calm.
(b) Multimodal analgesia (MMA) program: Two hours before surgery, nonsteroidal anti-inflammatory drugs (NSAIDs) (celecoxib 100 mg) and pain threshold raising drugs (pregabalin 75 mg) were taken orally. If there was no contraindication, then mid- and long-acting adrenocortical hormone (betamethasone 4 mg) was administered to the local nerves before the wound was closed, and oral NSAIDs (celecoxib), pain threshold-raising drugs (pregabalin), and topical analgesic plaster around the surgical area were used for postoperative analgesia.
(c) Individualized local anesthesia: Local anesthesia was used for all patients in DS group. Before surgery, patients with L5/S1 disc herniation were positioned prone, while patients with L3/4 and L4/5disc herniation were positioned laterally. The hierarchy of anesthesia via the posterior interlaminar approach is as follows: subcutaneous, fascia, the surface of ligamentum flavum, and the nerve peripheral, whereas the hierarchy of anesthesia via the lateral transforaminal approach is as follows: subcutaneous, fascia, ligamentum flavum surface, intervertebral foramen. Suppose the posterior interlaminar approach is used for patients with severe preoperative pain. In that case, inability to maintain position, severe intervertebral disc calcification, or massive disc herniation, the lateral intermorainal nerve block anesthesia should be used in addition to the level of anesthesia described above. At the end of the surgery, the dural sac and nerve-root were freely mobilized (Figure 1).
(d) Postoperative rehabilitation exercise: After surgery, the patient should actively perform rehabilitation exercises such as straight-leg-raising movement, ankle pump exercise, toe flexion, and extension. On the second postoperative day, the patient should wear a waist protector and ground exercise.

### Data Collection

The LOS, VAS for low back and leg pain, and ODI were recorded. All patients were followed up at immediately after surgery, one month, and one year postoperatively.

### Statistics

Measurement data with a normal distribution were expressed as mean ± standard deviation $(\bar{X}\pm S)$, and the paired t-test was used to compare before and after treatment. The independent t-test was used to compare groups; count data were expressed as a rate or composition ratio, and the comparison between groups was performed using the *χ*^2^ test. SPSS 23.0 software was used for statistical processing, and *p* < 0.05 was considered statistically significant.

## Results

A total of 290 patients were included in the follow-up according to the inclusion criteria, fifteen patients were lost to follow up for various reasons (e.g., immigration abroad, accidents, out of touch), and 275 patients finally completed the follow-up. The DS group had 152 patients treated by F-PELD combined with ERAS, with 104 males (68.4%) and 48 females (31.6%), and the average age of DS group was 36.53 ± 12.52 years old. In the TS group, 123 patients were treated by F-PELD combined with the traditional ideas, with 73 males (60.3%) and 50 females (40.7%), and the average age of TS group was 36.14 ± 11.98 years old. There was no statistically significant difference in sex ratio (*p* = 0.119), average age (*p* = 0.735), BMI (*p* = 0.693) between the two groups. However, the difference in LOS between these two groups was statistically significant. LOS of DS group was 1.38 ± 0.49 days, and DS group was 5.83 ± 2.24 days (*p* < 0.001). some postoperative complications occurred in TS group, including throat discomfort (*n* = 5, 4.1%), discomfort after catheterization (*n* = 7, 5.7%), abdominal distention (*n* = 3, 2.4%), and nausea (*n* = 5, 4.1%), none of which had serious consequences. After symptomatic treatment, all patients with complications improved within 72 h. Unlike TS group, DS group did not have these complications (Table 1).

**Table 1:** General characteristics.

	DS group	TS group	*p*–value
N	152	123	–
Male/female	104/48	73/50	0.119
Age(years)	36.53 ± 12.52 (24–48)	36.14 ± 11.98 (25–48)	0.735
Levels involved			
L3-L4	1 (0.7%)	3 (2.4%)	0.325
L4-L5	75 (49.3%)	59 (48.0%)	
L5-S1	73 (48.0%)	61 (49.6%)	
Two-segment	3 (2.0%)	–	
BMI	24.92 ± 3.75	27.74 ± 3.51	0.693
LOS(days)	1.38 ± 0.49	5.83 ± 2.24	<0.001*
Postoperative symptoms			
Throat sore	–	5 (4.1%)	–
Bloating	–	3 (2.4%)	–
Disgusting	–	5 (4.1%)	–
Post-catheterization discomfort	–	7 (5.7%)	–

*: *The difference is statistically significant.*

**Figure 2:**
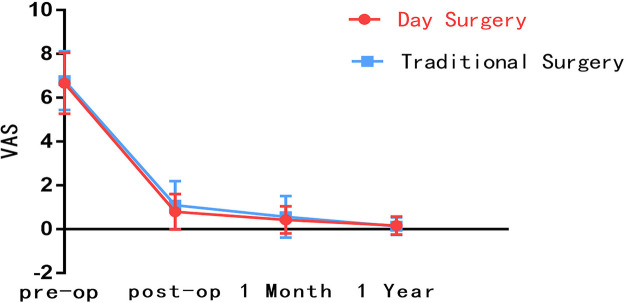
Comparison of VAS for back pain between DS group and TS group.

**Figure 3:**
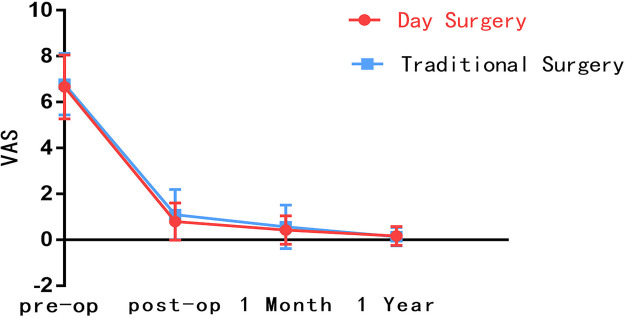
Comparison of VAS for leg pain between DS group and TS group.

**Figure 4:**
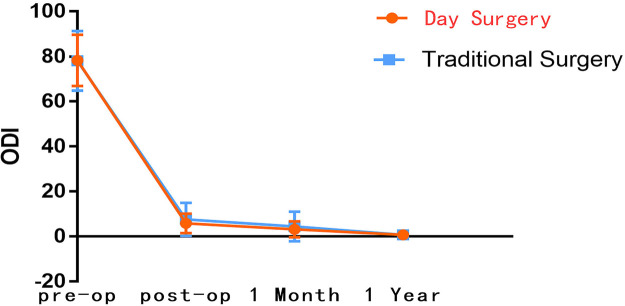
Comparison of ODI between DS group and TS group.

Preoperative VAS for back pain was recorded (5.9 ± 1.9) in DS group and (5.8 ± 2.0) in the TS group, *p* = 0.668. Preoperative VAS for leg pain was recorded (6.7 ± 1.4) in DS group and (6.8 ± 1.3) in TS group, with *p* = 0.486. Similarly, we recorded the preoperative ODI between DS group (78.3 ± 11.3) and TS group (78.1 ± 13.2), *p* = 0.878. We could not see a statistically significant difference between the two groups for preoperative VAS for leg pain (*p* = 0.668), preoperative VAS for back pain (*p* = 0.486), and ODI (*p* = 0.878). However, statistically significant differences were found in immediately postoperative VAS (back pain: DS group 1.4 ± 1.1, TS group 2.0 ± 1.2, *p* < 0.001; leg pain: DS group 0.8 ± 0.8, TS group 1.1 ± 1.1, *p* = 0.010) and one-month postoperative VAS (back pain: DS group 0.8 ± 0.9, TS group 1.1 ± 1.0, *p* = 0.035) were statistically significant differences. Similarly, statistically significant differences were also found in immediately postoperative ODI (DS group 5.8 ± 4.3, TS group 7.6 ± 7.4, *p* = 0.010) and one-month postoperative ODI (DS group 3.2 ± 3.5, TS group 4.5 ± 6.5, *p* = 0.036). Interestingly, the subsequent follow-up showed that this was not the case. The VAS (back pain: DS group 0.3 ± 0.6, TS group 0.3 ± 0.7, *p* = 0.798; leg pain: DS group 0.2 ± 0.4, TS group 0.1 ± 0.4, *p* = 0.485) and ODI (DS group 0.8 ± 1.2, TS group 0.7 ± 1.7, *p* = 0.729) was recorded one year postoperatively with improved postoperative pain and dyskinesia in both groups. However, no statistically significant difference was found between the two groups (Tables 2–4). The follow-up data indicate that DS group had the maximal differences compared with TS group at immediate post-operation, and ERAS provided significant advantages. As the patient recovered, the differences between the two groups shrank, and the benefits of ERAS began to fade (Figures 2–4).

**Table 2.** Comparison of VAS for back pain between DS group and TS group.

Follow-up time	DS group $(\bar{X}\pm S)$	TS group $(\bar{X}\pm S)$	*p* value
Preoperative	5.9 ± 1.9	5.8 ± 2.0	0.668
Immediately postoperative	1.4 ± 1.1	2.0 ± 1.2	<0.001*
One-month postoperative	0.8 ± 0.9	1.1 ± 1.0	0.035*
One-year postoperative	0.3 ± 0.6	0.3 ± 0.7	0.798

*: *The difference is statistically significant.*

**Table 3.** Comparison of VAS for leg pain between DS group and TS group.

Follow-up time	DS group $(\bar{X}\pm S)$	TS group $(\bar{X}\pm S)$	*p* value
preoperative	6.7 ± 1.4	6.8 ± 1.3	0.486
Immediately postoperative	0.8 ± 0.8	1.1 ± 1.1	0.010*
One-month postoperative	0.4 ± 0.6	0.6 ± 1.0	0.144
One-year postoperative	0.2 ± 0.4	0.1 ± 0.4	0.485

*: *The difference is statistically significant.*

**Table 4:** Comparison of ODI between DS group and TS group.

Follow-up time	DS group $(\bar{X}\pm S)$	TS group $(\bar{X}\pm S)$	*p* value
preoperative	78.3 ± 11.3	78.1 ± 13.2	0.878
Immediately postoperative	5.8 ± 4.3	7.6 ± 7.4	0.010*
One-month postoperative	3.2 ± 3.5	4.5 ± 6.5	0.036*
One-year postoperative	0.8 ± 1.2	0.7 ± 1.7	0.729

*: *The difference is statistically significant.*

## Discussion

Day surgery is a safe and dependable operation mode that involves selecting suitable patients and arranging hospitalization, surgery, short-term postoperative observation, and discharge from the hospital within 1–2 working days (17). Day surgery has two modes. The first mode of day surgery entails discharge on the same day as the procedure, with a 2–6 h postoperative observation period. Another option is overnight observation, with discharge on the first day after surgery if the total observation time is less than 24 h or the LOS is less than 48 h. ERAS, which advocates a series of perioperative optimization measures such as preoperative education, shorter abrosia time, MMA, individualized anesthesia program, intraoperative temperature, fluid management, and postoperative rehabilitation exercise, has recently become widely used in surgery. In contrast, traditional surgery may be more conservative than the ERAS concept, such as longer abrosia time, more frequent gastrointestinal decompression, catheterization, and general anesthesia, leading to several postoperative complications and discomfort. Thus, ERAS is well represented in the DS mode. Previous research concluded that combining DS with ERAS significantly reduced LOS, relieved perioperative physical and psychological stress, reduced perioperative complications, and produced a better clinical effect (18–20). As a result, the new spinal DS mode, which combined ERAS with minimally invasive spinal surgery, was widely used to treat spinal disease to achieve a safe, minimally invasive, and efficient result. For example, F-PELD combined with the ERAS concept to achieve the DS mode to treat LDH. However, few studies have been conducted to determine whether the DS mode has better short-term and long-term postoperative effects than the TS mode.

The purpose of this study was to see if F-PELD with DS mode produced better clinical results than F-PELD with TS mode. The results show that implementing DS reduced the LOS from 5.83 ± 2.24 days for day surgery to 1.38 ± 0.49 days, allowing patients to return to normal life and considerably improving the ward turnover rate. Compared to the TS group, the DS group can treat 2–3 times as many patients simultaneously. It was assisting more patients earlier in relieving pain caused by LDH. However, ERAS considers not only the improvement but also the recovery. As a result, VAS and ODI are used to assess the clinical efficacy of these two surgery modes. The findings suggested that there were no significant statistical differences in preoperative VAS between two groups. But the VAS and ODI at immediate post-operation (VAS for back pain: DS group 1.4 ± 1.1, TS group 2.0 ± 1.2, *p* < 0.001; VAS for leg pain: DS group 0.8 ± 0.8, TS group 1.1 ± 1.1, *p* = 0.010; ODI: DS group 5.8 ± 4.3, TS group 7.6 ± 7.4, *p* = 0.010) and one-month after surgery (VAS for back pain: DS group 0.8 ± 0.9, TS group 1.1 ± 1.0, *p* = 0.035; ODI: DS group 3.2 ± 3.5, TS group 4.5 ± 6.5, *p* = 0.036) had significant statistical differences. These results suggested that postoperative pain and dyskinesia were improved in both groups, and F-PELD in DS group could produce a better short-term effect. Interestingly, the VAS and ODI were further improved at one year after surgery, but no statistically significant difference was found between the two groups. It implies that the differences between the two groups will gradually narrow as the patient recovers and F-PELD in DS group produced a similar clinical effect compared with TS group at one year postoperatively. After surgery, ERAS plays a critical role in improving symptoms and enhancing recovery in the short term. However, more research into the long-term effects is required. Furthermore, throat discomfort (*n* = 5, 4.1%), discomfort after catheterization (*n* = 7, 5.7%), abdominal distention (*n* = 3, 2.4%), and nausea (*n* = 5, 4.1%) occurred in the TS group as postoperative complications, but none of the above complications happened in the DS group. In a word, the F-PELD in DS mode following ERAS should be promoted more widely due to these significant advantages in promoting rapid recovery, improving patients experience and shortening LOS.

This finding may change the perception that day surgery has a better clinical effect than traditional surgery. We believe that there are several reasons for the above results. Research has shown that stress, anxiety, and other negative emotions can lower the pain threshold. Preoperative education and optimized perioperative management were advocated by ERAS to make patients more relaxed and comfortable perioperatively, improving patient compliance and experience, assisting patients in tolerating postoperative discomfort, and lowering pain score and disability index. Also, the MMA program of ERAS effectively alleviates perioperative pain, allowing patients to exercise and return to normal life, increasing the efficiency of functional exercise, and assisting with patient recovery (21–28). Finally, the outcomes of F-PELD performed under local anesthesia are satisfactory. Local anesthesia not only reduces preoperative preparation time and requirements but also reduces postoperative recovery time. All patients in the DS group were given local anesthesia with no need for catheterization, preoperative intestinal preparation, or anything else. As a result, they experience less postoperative pain (29–31). This is also why patients in the TS group experience throat discomfort, discomfort after catheterization, abdominal distention, and nausea, whereas none of the aforementioned complications occur in the DS group. These patients typically have severe nerve root compression for calcification of disc herniation and massive disc herniation. These patients may experience severe pain if the surgeon detects and decompresses the nerve root. Furthermore, two obese patients experienced severe pain during surgery. Factors such as calcification of disc herniation, massive disc herniation, or obesity could cause severe pain in patients during surgery (32–34). Thus, we recommend that the surgeon use posterior local anesthesia combined with lateral foraminal area infiltration anesthesia or directly general anesthesia by experienced doctors when using the posterior interlaminar approach.

This research has some limitations. All the TS were carried out in 2019, but the DS was performed in 2020, so there may be influences of the surgical team’s tacit cooperation and experience. Furthermore, this study is a retrospective study, which inevitably produces bias in patient selection, information acquisition and other aspects.

## Conclusions

In combination with ERAS, F-PELD provides an effective day surgery mode for LDH. The day surgery with ERAS produced more satisfactory short-term clinical effects and reduced LOS, which promoted the rapid postoperative recovery of patients and accelerated turnover efficiency, and which is worthy of promotion.

## Data Availability

The original contributions presented in the study are included in the article/Supplementary Material, further inquiries can be directed to the corresponding author/s.
